# Exploring the reliability and profile of frequent mental health presentations using different methods: An observational study using statewide ambulance data over a 4-year period

**DOI:** 10.1177/00048674241289016

**Published:** 2024-10-26

**Authors:** Anthony Hew, Jesse T Young, Bosco Rowland, Debbie Scott, Ziad Nehme, Shalini Arunogiri, Dan I Lubman

**Affiliations:** 1 Monash Addiction Research Centre, Eastern Health Clinical School, Monash University, Melbourne, VIC, Australia; 2 Institute for Mental Health Policy Research, Centre for Addiction and Mental Health, Toronto, ON, Canada; 3 Dalla Lana School of Public Health, University of Toronto, Toronto, ON, Canada; 4 Centre for Epidemiology and Biostatistics, Melbourne School of Population and Global Health, The University of Melbourne, Parkville, VIC, Australia; 5 Centre for Adolescent Health, Murdoch Children’s Research Institute, Parkville, VIC, Australia; 6 School of Population and Global Health, The University of Western Australia, Perth, WA, Australia; 7 Justice Health Group, Curtin School of Population Health, Curtin University, Perth, WA, Australia; 8 Turning Point, Eastern Health, Melbourne, VIC, Australia; 9 Centre for Social and Early Emotional Development, School of Psychology, Faculty of Health, Deakin University, Geelong, VIC, Australia; 10 Australia Institute for Health Transformation, Deakin University, Geelong, VIC, Australia; 11 GLOBE, School of Health and Social Development, Deakin University, Geelong, VIC, Australia; 12 Centre for Research and Evaluation, Ambulance Victoria, Blackburn North, VIC, Australia; 13 Department of Epidemiology and Preventive Medicine, Monash University, Melbourne, VIC, Australia; 14 Department of Paramedicine, Monash University, Melbourne, VIC, Australia

**Keywords:** Ambulance, emergency medical services, frequent presenters, repeat presenters, frequent service use, emergency mental health, emergency services

## Abstract

**Introduction:**

A disproportionate number of mental health presentations to emergency services are made by frequent presenters. No current consensus definition of a frequent presenter exists. Using a statewide population-based ambulance database, this study (i) applied previous statistical methods to determine thresholds for frequent presenters, (ii) explored characteristics of the identified frequent presenter groups compared to non-frequent presenters and (iii) assessed the reliability of these methods in predicting continued frequent presenter status over time.

**Methods:**

Statistical methods utilised in previous studies to identify frequent presenters were applied to all ambulance attendances for mental health symptoms, self-harm and alcohol and other drug issues between 1 January 2017 and 31 December 2020 in Victoria, Australia. Differences in characteristics between identified frequent and non-frequent presenter groups were determined by logistic regression analysis. The consistency of agreement of frequent presenter status over time was assessed using intraclass correlation coefficients.

**Results:**

Thresholds for frequent presenters ranged from a mean of 5 to 39 attendances per calendar year, with groups differing in size, service use and characteristics. Compared to non-frequent presenters, frequent presenters had greater odds of being female, presenting with self-harm, experiencing social disadvantage or housing issues, involving police co-attendance and being transported to hospital. All frequent presenter definitions had poor reliability in predicting ongoing frequent presentations over time.

**Conclusion:**

A range of methods can define frequent presenters according to thresholds of yearly service use. Reasons for identifying frequent presenters may influence the method chosen. Future studies should explore definitions that capture the dynamic nature of presentations by this group.

## Introduction

Over the past decade, mental health-related presentations to emergency settings including emergency departments (EDs) and ambulance services (also known as Emergency Medical Services (EMS)) have increased by up to 30%, both in Australia (AIHW, 2023; Brazel et al., 2023; Lowthian et al., 2011). and internationally (Larkin et al., 2006; Lavergne et al., 2022: 2; Santillanes et al., 2020). A disproportionate number of these acute mental health presentations relate to a small group of individuals with repeated or frequent attendances (Barratt et al., 2016; Kromka and Simpson, 2019; Schmidt, 2018; Scott et al., 2014; Vandyk et al., 2013) and consume considerable resources from acute services (Ruger et al., 2004). In ambulance settings, frequent presenters have been found to comprise 0.2% to 23% of those receiving care and 1% to 40% of total ambulance transports (Scott et al., 2014).

Have been limited by several methodological issues. A key limitation of research into frequent presenters is the lack of consensus on how this group should be defined. In the literature, frequent presenters are commonly conceptualised as individuals who present over a certain threshold during a 1-year time frame. These thresholds have ranged from 3 to 20 or more presentations per year in emergency settings (Giannouchos et al., 2019; Krieg et al., 2016; LaCalle and Rabin, 2010; Soril et al., 2016; Vandyk et al., 2013). The lack of a consistent definition of frequent presenters has resulted in considerable variations in the size, patient characteristics and service demand of this group (Shannon et al., 2020), making it difficult to compare or aggregate findings across studies (Krieg et al., 2016; Pines et al., 2011).

The most common definition of a frequent presenter for mental health reasons is 4 or 5 attendances in a 1-year period (Schmidt, 2018; Vandyk et al., 2013). Many studies have arbitrarily applied this definition, despite differences in demographics, setting and location of their study population. A more rigorous and reliable approach would be to apply empirical approaches that use the distribution and clustering of study population data to determine classification (Beck et al., 2016; Scott et al., 2023; Suesse et al., 2023). Examples of approaches used to define frequent presenters in the literature include simple statistical methods that identify individuals who are among the top proportion of service users,. Example include those contributing to the top 2% (Beck et al., 2016), 5% (Ngamini-Ngui et al., 2014), 10% (Levola et al., 2019; Saarento et al., 1998) or 20% (Giannouchos et al., 2019; LaCalle and Rabin, 2010) of presentations each year, or over two standard deviations above the population mean (Beck et al., 2016; Pasic et al., 2005). More complex statistical methods have applied probabilistic modelling techniques (e.g. Poisson or negative binomial mixture modelling) to identify a cut-point number of presentations made by a sub-group of frequent presenters within the overall population (Beck et al., 2016; Locker et al., 2007; Suesse et al., 2023).

Few studies have explored and compared the different methods of identifying frequent presenters and the resulting size, attendance patterns, and characteristics of the groups. Given the range of methods used to identify and define frequent presenters, there is a need to understand how frequent presenters may differ according to the definition used. In addition, few studies have explored the longer-term trajectories of frequent presenters, with the existing research finding that only a minority of frequent presenters have repeated presentations beyond a one-year period (Kennedy and Ardagh, 2004; Lago et al., 2019).

To date, research into frequent presenters has focused on hospital in-patient and ED settings with a paucity of research into mental health-related frequent attendances to ambulance services (Roggenkamp et al., 2018). This is an important area of study given Australia has seen a 29% increase in ambulance attendances over the past decade, with the greatest growth in demand being attributed to attendances for mental health and alcohol and other drug (AOD)-issues (Andrew et al., 2020). Frequent presenters to ambulance services are over-represented by vulnerable individuals with complex needs (Snooks et al., 2019), who present with mental and behavioural problems including suicidal behaviour, and substance use issues (Scott et al., 2014). To date, there has been a paucity of research focused on frequent presentations for mental-health reasons to ambulance services.

Ambulance attendances provide a unique perspective on health system demands, as they offer coverage across entire states or territories including regional areas, can occur in public and private settings, and can also involve acute presentations that do not result in transportation to hospital. Paramedic records often include additional contextual information at the scene, such as if police also attended (Every-Palmer et al., 2023). Presentations to ambulance services may be of higher acuity or severity compared to those in an ED (Every-Palmer et al., 2023) or hospital setting (Wardrop et al., 2023).

Using a statewide population-based dataset of mental-health-related ambulance attendances, this study aimed to (i) apply a range of previously applied statistical methods to determine the thresholds of attendances in a 1-year period that define a frequent presenter; (ii) explore characteristics of the identified frequent presenter groups, with comparison to non-frequent presenters; and (iii) assess the reliability of these methods in identifying continued frequent presenter status over time.

## Methods

### Data source

This was an observational study using data derived from the Victorian arm of the National Ambulance Surveillance System (NASS). The NASS dataset contains ambulance records filtered for attendances where mental health, self-harm, or alcohol or other drugs were a significant or contributing factor to the attendance. Trained researchers then review and code the electronic paramedic records (ePCRs), which contain information taken from paramedic clinical assessments, patient self-report, information from third parties (e.g. family or bystanders) and evidence or observations at the scene. The methodology has been detailed in previous articles (Lubman et al., 2020; Scott et al., 2020).

We included for analysis records of all Victorian ambulance attendances to individuals aged 15 to 65 inclusive from 1 January 2017 to 31 December 2020. Victoria is Australia’s second most populous state, with a total population of over 6 million people (Australian Bureau of Statistics, 2021).

Each patient was assigned a unique Statistical Linkage Key (SLK-581) comprising 14 characters generated from a concatenation of components of family and given names (5 characters), the patient’s date of birth (8 characters) and gender (1 character). This allowed repeat presentations by the same patient to be identified in a privacy-preserving manner (AIHW, 2005). Attendances with missing or incomplete SLK-581 were excluded from analyses.

Ethics approval for the project was provided by Eastern Health and Ambulance Victoria.

### Classification of mental health-related ambulance attendances

The mental health-related ambulance attendances were categorised into psychiatric symptoms, self-harm and/or AOD issues. These were not mutually exclusive and individual cases could be coded as one or more of these categories. These categories were coded as binary variables (value of 0 if not present, value of 1 if present).

Psychiatric symptoms included attendances where there were symptoms of the following sub-categories: (a) anxiety, (b) depression, (c) psychosis, (d) social and emotional distress or (e) other unspecified mental health symptoms. Psychiatric symptoms that were due to an underlying acute physical cause (e.g. effects of an acute traumatic brain injury) were not included.

Attendances for self-harm included cases involving both self-injury and suicide-related presentations such as acts of self-injury, suicidal ideation, suicide attempt and/or suicidal acts. Where there was an unknown reason for overdose, these attendances were categorised as an AOD issue instead of self-harm as the intent of the overdose was unknown.

Attendances for AOD issues included cases where recent (in the past 24 hours) over or inappropriate use of (a) alcohol, (b) illicit drug/s, (c) pharmaceutical medications contributed to the ambulance attendance. For cases where substances were consumed, but the type of substance was not known, this was coded as (d) unknown substances.

Further specifications and inclusion criteria for the above are presented in Supplementary Appendix 1.

### Variables

Variables collected from the NASS database were categorised into binary variables, coded as either being present (value of 1) or not present (value of 0). The variables included (i) gender of the patient (only male and female gender were included for analysis due to the small counts of genders other than male or female); (ii) age group of individuals: 15 to 24, 25 to 44 and 45 to 65, which correspond to the age groups serviced by youth and adult mental health services in Victoria (Victorian Department of Health, 2024) and which have also been applied in a research context (Dinh et al., 2016; Krieg et al., 2016); (iii) geographic location (metropolitan or rural); (iv) day of week; (v) if emergency mental health services were involved or provided input for the case (either on the scene or via phone); (vi) if police co-attended; (vii) if the attended person was transported to hospital; (viii) the time of attendance (daytime – 08:00 to 15:59, evening – 16:00 to 23:59 or night – 00:00 to 07:59 hours); (ix) quintile rating ( 1 to 5) for relative social disadvantage according to the patient’s postcode of residence according as defined by the SocioEconomic Indexes For Areas Index of Relative Socio-economic Disadvantage (SEIFA–IRSD) 2016 (Australian Bureau of Statistics, 2018) where a lower quintile rating indicated an area of greater socioeconomic disadvantage compared to an area with a higher rating; (x) no recorded residential postcode if the person did not have a usual area of residence at the time of attendance or was unable to provide this to paramedics or; (xi) housing problems if individuals reported experiencing issues with homelessness, temporary accommodation (e.g. couch surfing, boarding house or homelessness accommodation) or stressors related to housing (e.g. eviction, overcrowding, poor quality housing).

### Analysis

To identify frequent presenter groups, the following statistical methods were applied to our study population to determine a threshold number of attendances for each calendar year:

1. The top 2% (Beck et al., 2016), 5% (Ngamini-Ngui et al., 2014), 10% (Levola et al., 2019; Saarento et al., 1998) or 20% (Giannouchos et al., 2019; LaCalle and Rabin, 2010) of the total ambulance attendances;
2. Two standard deviations (two SDs) above the total population mean (Beck et al., 2016); and
3. Identifying latent classes through applying mixture distribution models including the negative binomial (Suesse et al., 2023), Poisson (Beck et al., 2016; Locker et al., 2007; Suesse et al., 2023) and zero-truncated Poisson (Suesse et al., 2023) models. These assume underlying distributions or latent classes of attendances that are due to frequent presenter groups and unlikely due to independent, random events. The posterior probability threshold was set at 95% with Akaike information criterion (AIC) and Bayesian information criterion (BIC) values were used to compare and select the best-fitting count data model.

The resulting thresholds were then averaged over each calendar year in the study period to derive a mean threshold of attendances.

The mean number of individuals in the identified frequent presenter groups over the study period, along with service use and demographic and presentation characteristics (as a proportion of total number of attendances) were calculated.

Binary logistic regression models were used to obtain odds ratios to examine the differences in demographic and clinical characteristics factors between the identified frequent presenter groups and non-frequent presenters for each calendar year. Clustered standard error estimators were applied to account for within-group correlation to the number of attendances. The odds ratios were presented as a range (min-max) across the study period.

To explore the long-term trajectory of frequent presenters and whether they continue to have patterns of frequent presentations over time, we determined the proportion of each frequent presenter group who remained frequent presenters over subsequent years. The reliability in which frequent presenter status predicted continued frequent presenter status over time was assessed using intraclass correlation coefficients (ICCs). ICCs and their 95% confidence intervals were calculated using a two-way random effects model to evaluate the consistency of agreement of frequent presenter status across consecutive calendar years. ICC values less than 0.5, between 0.5 and 0.75, between 0.75 and 0.9, and greater than 0.90 were classified as poor, moderate, good, and excellent reliability, respectively (Koo and Li, 2016).

All statistical analysis was conducted using Stata version 17 (StataCorp, 2021).

## Results

There were 305,695 ambulance cases for mental health-related reasons over the four-year study period. We excluded 6887 (2.3%) records that had missing or incomplete SLK-581 identifiers. A total of 298,808 (97.7%) records were included in the study. A mean of 74,702 attendances were made to 50,366 individuals each calendar year. Both the total attendances and the attended individuals numerically increased over time, as shown in Table 1. Most individuals (80.3%) only had a single attendance made each calendar year. There was a median of 1 attendance (Interquartile range, IQR between 1–3 and 1–4) and a mean of 4.7 attendances (standard deviation, SD 11.0) to each patient per calendar year. An average of 14 individuals each year (0.03% of individuals) had 50 or more attendances (1.3% of all attendances).

**Table 1.** Summary statistics of ambulance attendances for mental-health-related reasons per calendar year.

	2017	2018	2019	2020	*Average of all years*
Attendances	65,583	71,188	78,512	83,525	74,702
*Mean (SD)*	5.0 (11.4)	4.6 (11.4)	4.3 (9.5)	5.0 (11.6)	4.7 (11.0)
*Median (IQR)*	1 (1–4)	1 (1–3)	1 (1–3)	1 (1–4)	1 (1–3 to 1–4)
*Max*	117	124	96	134	111
Individuals	43,610	49,516	53,731	54,607	50, 366

IQR: interquartile range.

### Defining frequent presenter groups

Using the previously reported identification methods, the resulting thresholds of attendances for frequent presenter groups are presented in Table 2. The mean thresholds for individual years presented in Table 3. Thresholds ranged from 39 (SD 4.0) or more attendances per year for the top 2% method to 5 (SD 0.58) or more attendances per year for the top 20% approach. Of the finite mixture modelling approaches, the truncated Poisson distribution model had the best fit according to AIC and BIC values (Table 2).

**Table 2.** Threshold number of attendances to define frequent presenter groups for each calendar year according to identification method.

Frequent presenter groups	2017	2018	2019	2020
Identification method				
*Top 2%*	41	41	33	41
*Top 5%*	22	18	17	22
*Top 10%*	11	9	9	11
*Top 20%*	5	4	4	5
*Two standard deviations*	28	28	24	29
*Poisson regression*	33	38	30	34
*AIC*	390,206	405,552.7	423,786.7	506476.2
*BIC*	390,233.3	405,580.2	423,814.5	506504.2
*Negative binomial regression*	Convergence not achieved	Convergence not achieved	Convergence not achieved	Convergence not achieved
*Truncated Poisson regression*	31	35	28	31
*AIC*	373,893.2	386,414.9	401,081.3	487519.3
*BIC*	373,920.5	386,442.4	401,109.1	487547.3

**Table 3.** Mean attendance thresholds, total yearly attendances and number of individuals among frequent presenter groups for the study period as determined by identification method.

Frequent presenter groups by identification method						
Mean for all years	Top 2%	Truncated Poisson	2 SDs	Top 5%	Top 10%	Top 20%
Threshold of attendances	39 (4.0)	32 (2.9)	27 (2.2)	20 (2.6)	10 (1.2)	5 (0.6)
Attendances (% of total)	1539 (2.1%)	2151 (2.9%)	2640 (3.5%)	3961 (5.3%)	7657 (10.3%)	16163 (21.6%)
Individuals (% of total)	27 (0.05%)	45 (0.09%)	61 (0.1%)	118 (0.2%)	418 (0.8%)	1928 (3.8%)

Presented as mean (range) or mean (%) for all years.

Defining frequent presenters using the top 2%, top 5%, top 2 standard deviation and truncated Poisson methods resulted in a small group of individuals (mean of 27 to 118 individuals per calendar year). Although these groups comprised 0.2% or less of all individuals, they contributed between 2.1% to 5.3% of yearly attendances. The top 10% and top 20% method resulted in larger frequent presenter groups (mean of 418 to 1928 individuals) comprising 0.8% and 3.8% of all attended individuals who contributed to a mean of 10.8% to 22.7% of attendances per calendar year.

### Characteristics of frequent presenter groups and comparing this to non-frequent presenters

The mean proportion and range of odds ratios (OR) for characteristics of the frequent presenter groups over the study period are presented in Table 4, with results for individual calendar years presented in Supplementary Tables 1–4.

**Table 4.** The proportion and odds ratio of demographic and presentation characteristics of frequent presenter groups.

Frequent presenter groups							All individuals
Characteristics	Top 2%	Truncated Poisson	> 2 Std Dev	Top 5%	Top 10%	Top 20%	
Regional location	17.9%	16.5%	17.9%	17.4%	19.8%	21.0%	24.3%
	0.26–1.3	0.3–0.95	0.45–1.05	0.45–0.86	0.6–0.9	0.69–0.88	
Female gender	64.8%	64.6%	62.1%	58.0%	53.4%	49.9%	47.0%
	1.48–2.59	1.54–2.9	1.53–2.67	1.29–2.22	1.14–1.53	1.1–1.28	
Age group							
15–24	19.3%	22.2%	24.1%	23.7%	21.9%	21.0%	25.2%
	0.29–0.95	0.41–1.43	0.64–1.7	0.61–1.36	0.65–1.81	0.62–0.95	
25–44	47.0%	45.7%	42.7%	41.2%	43.0%	46.3%	45.5%
	0.7–1.94	0.76–1.4	0.69–1.23	0.75–1.05	0.86–0.98	0.99–1.13	
45–65	33.7%	32.0%	33.2%	35.1%	35.1%	32.7%	29.3%
	1.01–1.33	0.96–1.26	0.91–1	0.99–1.91	1.03–1.66	1.06–1.36	
Area of residence^a^							
SEIFA 1 ( most disadvantage)	34.1%	29.2%	28.4%	27.3%	25.3%	24.7%	24.6%
	0.88–2.28	0.9–1.86	0.87–1.64	0.89–1.54	0.91–1.16	0.91–1.03	
SEIFA 2	12.9%	13.7%	15.2%	15.5%	15.5%	15.4%	15.8%
	0.91–1.15	0.49–1.06	0.42–1.13	0.69–1.06	0.88–1.06	0.91–1.02	
SEIFA 3	12.2%	12.5%	12.9%	13.1%	13.9%	14.7%	16.5%
	0.71–1.01	0.62–1.01	0.58–1.1	0.63–1	0.73–0.92	0.77–0.9	
SEIFA 4	15.0%	16.0%	15.8%	15.7%	16.4%	16.7%	17.08%
	0.57–1.42	0.44–1.61	0.78–1.53	0.74–1.18	0.86–1.05	0.89–1.00	
SEIFA 5 (least disadvantage)	9.6%	10.1%	10.7%	14.6%	15.4%	15.5%	16.5%
	0.16–0.81	0.19–0.85	0.34–0.79	0.51–1.02	0.72–0.99	0.84–1.01	
Residence not recorded	15.9%	15.1%	14.6%	13.7%	13.5%	13.0%	9.5%
	1.05–3.5	1.02–2.82	1.09–2.56	1.13–2.35	1.45–2.17	1.79–1.2	
Housing problems	8.4%	7.7%	7.3%	7.0%	7.0%	6.7%	3.8%
	1.17–4.09	1.18–3.08	1.22–2.95	2.13–2.69	1.66–2.62	2.11–2.65	
Time of the day							
Day (08:00–16:59)	27.4%	28.3%	28.5%	28.4%	29.6%	31.0%	29.5%
	0.75–1.2	0.82–1.14	0.84–1.14	0.82–1.09	0.91–1.1	1.01–1.13	
Evening (17:00–23:59)	54.1%	54.5%	54.2%	54.3%	52.7%	50.6%	46.9%
	1.24–1.42	1.29–1.53	1.25–1.52	1.31–1.53	1.25–1.36	1.15–1.27	
Night (00:00–07:59)	18.5%	17.2%	17.2%	17.3%	17.7%	18.4%	23.6%
	0.56–0.85	0.52–0.79	0.5–0.79	0.53–0.77	0.56–374	0.61–0.78	
Weekend attendance	29.1%	28.6%	28.4%	27.6%	27.1%	27.2%	31.5%
	0.8–0.99	0.79–0.94	0.76–0.93	0.75–0.89	0.71–0.86	0.69–0.82	
Psychiatric symptoms^b^	24.7%	24.1%	23.8%	24.3%	28.2%	33.5%	36.4%
	0.51–0.73	0.48–0.63	0.5–0.58	0.45–0.64	0.59–0.69	0.84–0.92	
Anxiety	2.2%	2.1%	2.2%	3.3%	4.4%	5.8%	10.5%
	0.07–0.39	0.07–0.17	0.08–0.3	0.11–0.33	0.31–0.38	0.45–0.48	
Depression	3.9%	4.0%	4.1%	4.5%	4.7%	5.1%	5.7%
	0.48–0.83	0.59–0.77	0.57–0.92	0.73–0.81	0.77–0.83	0.81–0.89	
Psychosis symptoms	12.2%	10.9%	10.1%	9.1%	9.3%	9.9%	7.6%
	1.49–1.91	1.41–1.54	1.4–1.43	1–1.53	1.07–1.36	1.39–1.58	
Other MH Sx	6.9%	7.6%	7.8%	7.7%	10.1%	13.2%	12.9%
	0.39–0.59	0.48–0.68	0.52–0.62	0.49–0.57	0.59–0.77	0.86–1.15	0.28–0.88
AOD issues^c^	54.8%	56.3%	56.7%	59.2%	60.2%	59.8%	60.2%
	0.59–0.99	0.68–1.09	0.69–1.02	0.74–1.04	0.92–1.07	0.95–1.03	
Alcohol	33.9%	34.5%	35.7%	39.7%	39.0%	34.6%	32.7%
	0.78–1.49	0.8–1.76	0.9–1.49	1.14–1.62	1.28–1.53	1.05–1.26	
Illicit substance	7.7%	8.8%	8.8%	8.8%	11.3%	14.6%	14.2%
	0.24–0.55	0.23–0.51	0.26–0.41	0.2–0.4	0.27–0.52	0.7–0.75	
Pharmaceutical medications	12.9%	11.6%	10.6%	10.2%	9.7%	10.4%	8.2%
	0.92–1.65	0.89–1.84	1.11–1.51	0.92–1.31	0.88–1.07	0.89–1.06	
Unknown substance	2.4%	2.8%	2.6%	2.5%	2.3%	2.8%	3.11%
	0.65–0.77	0.59–0.77	0.61–0.85	0.47–0.73	0.65–0.81	0.89–1.00	
Self Harm^d^	66.2%	64.4%	62.8%	57.5%	51.5%	45.9%	37.1%
	2.48–4.30	2.2–3.8	2.45–3.44	2.16–2.82	1.89–2.04	1.56–1.69	
Transport to hospital	90.7%	90.9%	90.8%	89.4%	88.6%	87.3%	82.1%
	1.63–2.78	1.92–2.92	1.93–2.6	1.78–2.08	1.71–1.95	1.62–1.72	
Police attended	53.2%	52.3%	51.6%	48.4%	46.1%	45.5%	40.8%
	1.53–2.01	1.5–1.79	1.45–1.77	1.3–1.56	1.22–1.35	1.24–1.34	

Results presented as the mean proportion of attendances and range of odds ratios compared to non-frequent presenter groups (min and max) over the study period. Odds ratios calculated to account for within-person clustering.

a Area of Residence SEIFA rating – Quintile rating ( 1 to 5) of relative social disadvantage according to the patient’s postcode of residence, based on the SocioEconomic Indexes For Areas Index of Relative Socioeconomic Disadvantage. A lower quintile number indicates an area of greater socioeconomic disadvantage compared to an area with a higher score.

b Psychiatric symptoms – includes symptoms of anxiety, depression, psychosis, social and emotional distress, or other unspecified mental health symptoms.

c AOD Issues–cases where recent (in the past 24 hours) over or inappropriate use of alcohol, illicit drugs or pharmaceutical medications was a contributing reason for attendance.

d Self Harm – includes cases involving self-injury, suicidal ideation, suicide attempt or suicide.

Compared to non-frequent presenters, frequent presenters had increased odds of being female (ORs range = 1.10 to 2.59), with and also greater odds of attendances involving self-harm (ORs range 1.56 to 4.30). Self-harm was recorded in 46% to 67% of all frequent presenter attendances. The odds of attendances involving psychosis were increased among frequent presenters, but decreased for other mental health symptoms, such as anxiety. Between 50.6% and 54.5% of attendances to frequent presenters occurred in the evening, with the odds of this occurring being greater among this group compared to non-frequent presenters (ORs range 1.15 to 1.53).

Compared to non-frequent presenters, the frequent presenter groups had increased odds of attendances where individuals reported housing problems (ORs range 1.02 to 3.5) or did not have a place of residence recorded (ORs range 1.05 to 3.50). Between 87.3% and 90.9% of attendances to frequent presenters resulted in transport to hospital and 45.5% to 53.2% involved police co-attendance, with the odds of these events being greater for frequent presenter group compared to non-frequent presenter (ORs range = 1.22 to 2.01 for transport to hospital and ORs range 1.62 to 2.78 for police co-attendance).

### Consistency of agreement of identified frequent presenter status across threshold groups

As shown in Table 5, among frequent presenter groups, 35.1% to 56.3% of individuals remained a frequent presenter in the following year, reducing to 12.5% to 27.3% of the individuals after three consecutive years and 11% to 19% after four consecutive years. Frequent presenter status had poor consistency of agreement of frequent presenter status over time, with ICC values remaining less than 0.5 across time (range 0.32–0.48).

**Table 5.** Test–retest reliability of frequent presenter status over consecutive years assessed using Intraclass Correlation Coefficients (ICCs).

Frequent presenter group						
	Top 2%	Truncated Poisson	> 2 std dev	Top 5%	Top 10%	Top 20%
Individuals (n) in 2017	22	37	55	98	318	1396
*n* (%) in 2018	9 (40.9%)	13 (35.1%)	22 (40.0%)	42 (42.9%)	138 (43.4%)	550 (39.4%)
ICC (95% CI)	0.42 (0.40, 0.44)	0.40 (0.38, 0.42)	0.47 (0.45, 0.49)	0.41 (0.39, 0.43)	0.39 (0.37, 0.41)	0.37 (0.35, 0.39)
*n* (%) in 2018 & 2019	7 (31.8%)	9 (24.3%)	17 (30.9%)	28 (28.86%)	80 (25.2%)	294 (21.1%)
ICC (95% CI)	0.44 (0.42, 0.46)	0.43 (0.41, 0.45)	0.48 (0.46, 0.50)	0.41 (0.39, 0.43)	0.40 (0.38, 0.42)	0.34 (0.32, 0.36)
*n* (%) in 2018 & 2019 & 2020	6 (27.3%)	8 (21.6%)	12 (21.8%)	18 (18.4%)	47 (14.8%)	175 (12.5%)
ICC (95% CI)	0.42 (0.40, 0.45)	0.40 (0.38, 0.42)	0.45 (0.42, 0.47)	0.39 (0.37, 0.42)	0.39 (0.36, 0.41)	0.32 (0.3, 0.34)
Individuals (n) in 2018	22	32	48	110	418	2091
*n* (%) in 2019	10 (45.5%)	18 (56.3%)	26 (54.2%)	49 (44.5%)	175 (41.9%)	749 (35.8%)
ICC (95% CI)	0.45 (0.43 0.46)	0.48 (0.47, 0.50)	0.47 (0.46, 0.49)	0.42 (0.40, 0.44)	0.42 (0.40, 0.43)	0.37 (0.35, 0.39)
*n* (%) in 2019 & 2020	9 (40.9%)	12 (37.5%)	15 (31.3%)	24 (21.8%)	85 (20.3%)	334 (16.0%)
ICC (95% CI)	0.43 (0.41, 0.45)	0.43 (0.41, 0.45)	0.43 (0.41, 0.45)	0.41 (0.39, 0.43)	0.40 (0.38, 0.42)	0.33 (0.31, 0.35)
Individuals (n) in 2019	31	49	73	145	505	2413
*n* (%) in 2020	12 (38.7%)	21 (42.9%)	29 (39.7%)	53 (36.6%)	164 (32.5%)	688 (28.5%)
ICC (95% CI)	0.41 (0.40, 0.43)	0.42 (0.41, 0.44)	0.44 (0.42, 0.46)	0.44 (0.43, 0.46)	0.40 (0.38, 0.41)	0.36 (0.34, 0.38)

## Discussion

### The findings in context

In this study, we examined a range of previously applied statistical methods to explore the variability in thresholds that define frequent presenters for acute mental health reasons. These thresholds ranged from 5 to 39 or more attendances per year. The identified frequent presenter groups contributing approximately 2% to 22% of the total attendances and comprising 0.05% to 4.9% of all attended individuals.

The frequent presenter groups in our study population showed key differences in attendance characteristics compared to non-frequent presenters. These groups had greater odds of attendances involving self-harm and psychosis, with similar findings observed in frequent presenters in acute mental health settings (Casey et al., 2021; Kromka and Simpson, 2019; Lewis et al., 2019; Schmidt, 2018; Suesse et al., 2021). Compared to non-frequent presenters, attendances for frequent presenters had greater odds of occurring in the evening. This may be due to a build-up of issues and symptoms during the day that have not responded to other approaches, as well as growing concerns from family and/or treating clinicians (Meehan et al., 2021), resulting in escalation to emergency services after-hours. It may also reflect barriers to accessing after-hours treatment and support from primary care or community-based services (Vandyk et al., 2018, 2019) emphasising the need to consider the opening times of services and available resource allocation.

In keeping with characteristics of the frequent mental health presenter populations described in ED and psychiatric emergency rooms (Kaltsidis et al., 2021; Schmidt, 2018), we also found greater odds of frequent presenters experiencing housing stress, homelessness and residing in areas of socioeconomic disadvantage. The experience of social exclusion and socioeconomic hardship have previously been identified as contributing to repeated mental health presentations to acute care settings (Kromka and Simpson, 2019; Schmidt, 2018). This highlights the need for broader, systems-based approaches to care for individuals with repeated mental health-related emergency presentations. Beyond clinical treatment, it is crucial to address factors such as socioeconomic stability and access to secure housing.

Compared to non-frequent presenters, frequent presenters also had greater odds of police involvement and being transported to hospital. This may indicate increased clinical acuity and possible risks to self and others. It demonstrates that frequent presenters to ambulance services for mental health reasons are also frequent users of other emergency care services and disproportionately contributing to overall resource demands (Thompson et al., 2011).

Arbitrarily chosen thresholds for frequent service use are problematic, as they lack generalisability across different systems and settings servicing vastly different populations. Data-driven methods of empirically defining thresholds for frequent presenters account for variations in the distribution of events specific to the study population. For example, the best-fitting model for identifying the frequent presenter group in our study population was the truncated Poisson regression model. In contrast, studies examining acute mental health unit hospitalisations found the negative binomial distribution was the best-fitting model (Beck et al., 2016; Suesse et al., 2023). This may be due to differences in attendance distributions unique to the study setting and populations, such as an overdispersion of single events among attendances by ambulance compared to admissions to a mental health unit.

### Implications for policy and practice

Our study did not set out to propose a single method or threshold to define frequent presenters, but rather to demonstrate the range of statistical methods available and impact they have on the identification of frequent presenters. The choice of method used results in groups of varying sizes, service demands and characteristics being identified.

Deciding on the method to define frequent presenters will depend on the reasons and goals for identifying this cohort. These may include research purposes, planning a clinical intervention, allocating resources or determining a public health response.

If the goal of identifying frequent presenters is to provide population-level interventions that involve improvements or reform to existing services and systems, broader definitions such as the top 20% of attendances may be more advantageous. This will identify a target population of sufficient size to justify resource allocation and planning at a population level. However, a more restricted definition may be preferable for local services that aim to identify and provide intensive interventions for the very small number of individuals who are their highest service users. These individuals will likely require person-centred multidisciplinary approaches and interventions, such as care coordination and case management, which target complex health and social needs in an integrated fashion (Mao et al., 2023). The use of complex statistical identification methods may be more suited to research settings where variances in population distribution and clustering of attendances may need to be considered. These might have less utility in clinical settings where the use of simpler statistical methods can provide a more easily obtained and readily interpretable metric.

Even with consistent and standardised definitions of frequent presenters, there will remain challenges with the comparability of frequent presenter data across studies. For example, our study comprised a cohort of ambulance service users who have been selected for mental health reasons only. Our findings cannot be generalised to other study populations where the frequent presenter cohort may include individuals who also present for reasons other than mental health or who accessing ED rather than ambulance services. This highlights the importance of identifying frequent presenters based on a key question of interest for the target population and to limitations of applying results across studies.

### Areas for further study

Frequent presenter status appears to be an acute and dynamic process that changes with time, rather than being a static concept. When conceptualised according to a 1-year period, consistency of agreement of frequent presenter status over time was poor. Less than 56% of all frequent presenters to ambulance services continued to remain a frequent presenter the following year and less than 27% remained a frequent presenter over three consecutive years. These findings are consistent with research examining frequent presenters to EDs (Lago et al., 2019; Mandelberg et al., 2000) and to mental health in-patient units (Shafer, 2019). Together, these findings highlight how most people with frequent presentations have acute, rather than chronic patterns of acute care need. As such, the widely used definitions of frequent presenter behaviour based on one-year periods have limited reliability in predicting future frequent service use. In addition, defining frequent presenters according to one-year periods has limited clinical relevance, with the need to wait a year to identify these individuals before intervention can be provided.

Alternative definitions of frequent presenters that enable the earlier identification of individuals at imminent risk of increased attendances are needed. An example could be defining frequent presenters according to service use in one-month time periods (Scott et al., 2023; Wong et al., 2022) which could allow for more timely clinical responses tailored to the dynamic nature of attendances by this group. The relatively substantial proportion of individuals who exhibit a persistent pattern of repeated presentations over multiple years also warrants further study and clinical consideration. This group may have unique clinical needs and may benefit from targeted intervention and support given their persistent high frequency pattern of presentations.

### Strengths and limitations

Strengths of our study include the use of a unique representative population-based dataset comprising ambulance attendances that are not restricted to a site or region. The use of multiple consecutive years of data accounts for year-to-year variations in population sizes and attendance patterns, providing a more stable definition of frequent presenters compared to the use of one calendar year only. It also enabled longer-term variations in the number and pattern of attendances among frequent presenters to be explored.

The dataset comprised paramedic care records that were systematically reviewed and coded by research assistants. resulting in the accurate and consistent recording of mental health symptoms. The use of paramedic records captures presenting symptoms that may otherwise not be recorded or missed in other administrative datasets such as hospital (Sveticic et al., 2019) or ambulance dispatch records (Moore et al., 2023).

Limitations of our study include the generalisability of our findings to other ambulance services in Australia or internationally. Dynamic changes to the healthcare system and socio-political environment may impact the profile of ambulance attendance patterns both over time and across jurisdictions. For instance, during the study period, initiatives such as telehealth mental health triage systems were implemented in Victoria to reduce or divert emergency mental health presentations (Ambulance Victoria, 2021). Our study period also included presentations during the COVID-19 pandemic (2020–2021), which may have impacted the number and reasons for mental health-related attendances. Indeed, a time-series analysis reported an increase in mental health-related ambulance attendance in Victoria during the COVID-19 lockdown periods (Andrew et al., 2022).

In this study, we have focused only on ambulance attendance for mental health-related reasons. However, frequent presenters have complex needs, including physical health co-morbidities. As such, this population may have also had ambulance attendance for physical health reasons (Duggan et al., 2020; Edwards et al., 2015) during the study period, thereby underestimating their overall utilisation of emergency services.

## Conclusion

A range of statistical methods can be used to define frequent presenters according to thresholds of service use in a 1-year period. These empirical approaches result in a broad range of frequent presenter groups that vary in size and have disparate clinical characteristics. The choice of method used should be tailored to the reasons and goals for identifying frequent presenters. Current approaches to defining frequent presenters have poor reliability over time and alternative approaches to conceptualising the dynamic process in which frequent presentation behaviour arises should be considered in future studies.

## Supplemental Material

sj-docx-1-anp-10.1177_00048674241289016 – Supplemental material for Exploring the reliability and profile of frequent mental health presentations using different methods: An observational study using statewide ambulance data over a 4-year period Supplemental material, sj-docx-1-anp-10.1177_00048674241289016 for Exploring the reliability and profile of frequent mental health presentations using different methods: An observational study using statewide ambulance data over a 4-year period by Anthony Hew, Jesse T Young, Bosco Rowland, Debbie Scott, Ziad Nehme, Shalini Arunogiri and Dan I Lubman in Australian & New Zealand Journal of Psychiatry
