# Supplementary material for: Exploring the reliability and profile of frequent mental health presentations using different methods: An observational study using statewide ambulance data over a 4-year period
Source: Aust N Z J Psychiatry. 2024 Oct 26;59(1):74–85. doi: 10.1177/00048674241289016 (PMC11667961; doi:10.1177/00048674241289016)
Supplement: sj-docx-1-anp-10.1177_00048674241289016 – Supplemental material for Exploring the reliability and profile of frequent mental health presentations using different methods: An observational study using statewide ambulance data over a 4-year period [file sj-docx-1-anp-10.1177_00048674241289016.docx]

***Supplementary Material***

***Appendix 1***

***Definitions: Identification of mental health-related ambulance attendances***

Ambulance attendances were categorized as involving psychiatric symptoms, self-harm or AOD issues if the following presenting issues or symptoms were recorded by paramedics at the time of ambulance attendance:

Psychiatric symptoms:

(a) anxiety: overwhelming and intrusive worry, and/or panic attack symptom profile;

(b) depression: symptoms of a depression including low mood, feelings of hopelessness, despair, worthlessness, anhedonia, change in appetite or sleep;

(c) psychosis: presence of delusions and hallucinations; (d) social and emotional distress: where this was reported during or immediately before attendance;

(e) other unspecified mental health symptoms that do not fit into the previous categories, such as mania and paranoia (Lubman et al., 2020b).

Mental health symptoms which were likely due to an underlying acute physical cause (such as a traumatic brain injury) were excluded.

Self-harm:

(a) self-injury: intentional non-fatal injury without suicide intent;

(b) suicidal ideation: thoughts of killing oneself without acting on thoughts;

(c) suicide attempt: intentional non-fatal injury with suicidal intent;

(d) suicide: intentional fatal injury with suicide intent occurred before (in the past 24 hours) or during the ambulance attendance.

AOD issues:

a) alcohol: for cases where there has been over or inappropriate alcohol use contributed to the attendance, as assessed through proxy measures including paramedic clinical assessment or report of amount consumed by individual

b) illicit drugs: for cases where the patient has consumed illicit drugs such as methamphetamines, cannabis, MDMA/ecstasy and heroin

c) pharmaceutical medications: for cases where there has been overuse or inappropriate use of pharmaceutical medications including prescription medications such as antipsychotics, antidepressants, benzodiazepines, anticonvulsants, opioid replacement therapies and stimulants, as well as over the counter medications such as analgesics ( e.g. – paracetamol, anti-inflammatories) that had been taken contrary to prescriber or manufacturer instructions, d) unknown substances: for cases when a substance has been consumed, but the type of substance is unknown

Where multiple categories of AOD use were involved, these were all coded individually.

Information about AOD issues was taken from the clinical notes and includes the assessments by paramedics , patient self-report, information from third parties (e.g., family or bystanders), and evidence at the scene (e.g., alcohol-related products or drug paraphernalia).

**Supplementary Table 1 - The proportion and odds ratio (95% CI) of demographic and presentation characteristics of frequent presenter groups in 2017**

| **2017** | **Frequent Mental Health Presenter Cohorts** | | | | | | **All individuals** |
| --- | --- | --- | --- | --- | --- | --- | --- |
| **Characteristics** | **Top 2%** | **Truncated Poisson** | **> 2 Std Dev** | **Top 5%** | **Top 10%** | **Top 20%** |  |
| **Regional location** | 11.9% | 8.5% | 12.3% | 13.0% | 18.6% | 19.6% | 23.3% |
| **Regional location** | 0.44 (0.16 - 1.17) | 0.3 (0.12 - 0.77) | 0.45 (0.24 - 0.86) | 0.48 (0.29 - 0.8) | 0.73 (0.56 - 0.96) | 0.76 (0.66 - 0.89) |  |
|  | 56.9% | 57.7% | 58.6% | 57.2% | 50.2% | 49.2% | 47.3% |
| **Female gender** | 1.48 (0.62 - 3.57) | 1.54 (0.77 - 3.08) | 1.61 (0.9 - 2.86) | 1.52 (0.98 - 2.36) | 1.14 (0.88 - 1.48) | 1.1 (0.96 - 1.27) |  |
|  |  |  |  |  |  |  |  |
| **Age group** | 9.3% | 12.6% | 18.5% | 22.0% | 20.6% | 18.9% | 25.6% |
| **15 - 24** | 0.29 (0.07 - 1.29) | 0.41 (0.15 - 1.11) | 0.65 (0.33 - 1.28) | 0.81 (0.5 - 1.33) | 0.73 (0.54 - 1) | 0.62 (0.52 - 0.74) |  |
|  | 61.5% | 53.6% | 50.5% | 46.8% | 45.1% | 47.9% | 45.6% |
| **25 - 44** | 1.94 (0.79 - 4.74) | 1.4 (0.7 - 2.77) | 1.23 (0.69 - 2.17) | 1.05 (0.68 - 1.64) | 0.98 (0.75 - 1.28) | 1.13 (0.98 - 1.3) |  |
|  | 29.2% | 33.7% | 31.1% | 31.2% | 34.3% | 33.2% | 28.9% |
| **45 - 65** | 1.01 (0.39 - 2.62) | 1.26 (0.62 - 2.57) | 1.11 (0.61 - 2.05) | 1.13 (0.71 - 1.8) | 1.33 (1.01 - 1.74) | 1.3 (1.11 - 1.51) |  |
|  |  |  |  |  |  |  |  |
| **Area of residence ^$^** | 22.3% | 22.5% | 22.1% | 22.5% | 22.9% | 23.1% | 24.5% |
| **SEIFA 1 ( most disadvantage)** | 0.88 (0.38 - 2.07) | 0.9 (0.46 - 1.76) | 0.87 (0.49 - 1.54) | 0.89 (0.58 - 1.37) | 0.91 (0.7 - 1.18) | 0.91 (0.79 - 1.05) |  |
|  | 8.2% | 7.2% | 11.3% | 11.3% | 13.9% | 14.4% | 15.4% |
| **SEIFA 2** | 0.49 (0.2 - 1.16) | 0.42 (0.2 - 0.88) | 0.69 (0.39 - 1.24) | 0.69 (0.43 - 1.09) | 0.88 (0.67 - 1.16) | 0.91 (0.78 - 1.06) |  |
|  | 12.3% | 11.0% | 11.0% | 13.4% | 13.7% | 14.5% | 16.5% |
| **SEIFA 3** | 0.71 (0.27 - 1.84) | 0.62 (0.27 - 1.42) | 0.62 (0.31 - 1.24) | 0.78 (0.48 - 1.28) | 0.79 (0.58 - 1.06) | 0.83 (0.71 - 0.98) |  |
|  | 22.4% | 24.5% | 23.6% | 19.3% | 16.6% | 16.7% | 17.0% |
| **SEIFA 4** | 1.42 (0.65 - 3.1) | 1.61 (0.86 - 2.99) | 1.53 (0.9 - 2.62) | 1.18 (0.74 - 1.87) | 0.97 (0.71 - 1.32) | 0.97 (0.82 - 1.15) |  |
|  | 11.3% | 15.0% | 13.8% | 16.7% | 17.1% | 17.3% | 17.1% |
| **SEIFA 5 (least disadvantage)** | 0.61 (0.26 - 1.47) | 0.85 (0.44 - 1.64) | 0.77 (0.43 - 1.37) | 0.97 (0.63 - 1.48) | 0.99 (0.76 - 1.3) | 1.01 (0.87 - 1.18) |  |
|  | 23.4% | 19.7% | 18.2% | 16.7% | 15.7% | 13.9% | 8.4% |
| **Residence not recorded** | 3.5 (1.6 - 7.68) | 2.82 (1.43 - 5.56) | 2.56 (1.43 - 4.6) | 2.35 (1.48 - 3.73) | 2.29 (1.73 - 3.04) | 2.17 (1.83 - 2.57) |  |
|  | 15.6% | 12.2% | 11.6% | 10.6% | 9.7% | 8.7% | 4.5% |
| **Housing Problems** | 4.09 (1.96 - 8.5) | 3.08 (1.57 - 6.05) | 2.95 (1.65 - 5.26) | 2.69 (1.68 - 4.32) | 2.62 (1.95 - 3.53) | 2.65 (2.21 - 3.17) |  |
|  |  |  |  |  |  |  |  |
| **Time of the day** | 32.9% | 31.9% | 31.9% | 30.8% | 30.9% | 30.9% | 29.1% |
| **Day (08:00 - 16:59)** | 1.2 (0.88 - 1.63) | 1.14 (0.88 - 1.47) | 1.14 (0.93 - 1.42) | 1.09 (0.92 - 1.29) | 1.1 (0.99 - 1.22) | 1.11 (1.04 - 1.18) |  |
|  | 52.1% | 53.9% | 54.3% | 54.5% | 53.5% | 51.6% | 46.9% |
| **Evening (17:00 - 23:59)** | 1.24 (0.98 - 1.57) | 1.34 (1.09 - 1.63) | 1.36 (1.15 - 1.6) | 1.38 (1.22 - 1.57) | 1.34 (1.24 - 1.46) | 1.27 (1.21 - 1.34) |  |
|  | 15.0% | 14.2% | 13.8% | 14.6% | 15.6% | 17.4% | 23.9% |
| **Night (00:00 - 07:59)** | 0.56 (0.43 - 0.72) | 0.52 (0.41 - 0.66) | 0.5 (0.41 - 0.62) | 0.53 (0.45 - 0.63) | 0.56 (0.49 - 0.63) | 0.61 (0.57 - 0.66) |  |
|  | 28.3% | 28.0% | 27.5% | 27.3% | 26.6% | 26.8% | 32.9% |
| **Weekend attendance** | 0.8 (0.7 - 0.92) | 0.79 (0.69 - 0.89) | 0.76 (0.68 - 0.85) | 0.75 (0.69 - 0.83) | 0.71 (0.67 - 0.76) | 0.69 (0.66 - 0.72) |  |
|  | 22.5% | 20.9% | 21.7% | 23.5% | 25.1% | 30.1% | 35.1% |
| **Psychiatric symptoms ^%^** | 0.53 (0.31 - 0.91) | 0.48 (0.31 - 0.73) | 0.5 (0.36 - 0.7) | 0.55 (0.43 - 0.71) | 0.59 (0.51 - 0.68) | 0.75 (0.69 - 0.82) |  |
|  | 2.0% | 2.4% | 2.4% | 4.1% | 5.6% | 6.6% | 12.0% |
| **Anxiety** | 0.15 (0.1 - 0.22) | 0.18 (0.12 - 0.26) | 0.18 (0.13 - 0.25) | 0.3 (0.2 - 0.46) | 0.4 (0.31 - 0.52) | 0.46 (0.4 - 0.53) |  |
|  | 3.2% | 4.2% | 4.2% | 4.7% | 5.1% | 5.4% | 6.3% |
| **Depression** | 0.48 (0.27 - 0.84) | 0.63 (0.42 - 0.97) | 0.64 (0.45 - 0.9) | 0.72 (0.55 - 0.95) | 0.77 (0.65 - 0.92) | 0.81 (0.73 - 0.91) |  |
|  | 11.8% | 9.5% | 9.5% | 9.0% | 8.4% | 9.7% | 7.0% |
| **Psychosis Symptoms** | 1.79 (0.8 - 4) | 1.41 (0.69 - 2.88) | 1.4 (0.79 - 2.49) | 1.33 (0.85 - 2.08) | 1.24 (0.94 - 1.64) | 1.58 (1.37 - 1.83) |  |
|  | 7.1% | 6.2% | 6.8% | 6.9% | 7.3% | 10.3% | 11.4% |
| **Other MH Sx** | 0.59 (0.35 - 1) | 0.5 (0.32 - 0.8) | 0.56 (0.39 - 0.81) | 0.56 (0.42 - 0.74) | 0.59 (0.49 - 0.7) | 0.86 (0.78 - 0.95) |  |
|  | 60.8% | 63.0% | 61.6% | 61.9% | 62.6% | 61.7% | 61.1% |
| **AOD issues** ^§^ | 0.99 (0.53 - 1.84) | 1.09 (0.65 - 1.81) | 1.02 (0.67 - 1.56) | 1.04 (0.75 - 1.44) | 1.07 (0.88 - 1.31) | 1.03 (0.93 - 1.15) |  |
|  | 41.8% | 45.7% | 41.7% | 41.0% | 41.6% | 36.8% | 32.7% |
| **Alcohol** | 1.49 (0.73 - 3.03) | 1.76 (1 - 3.11) | 1.49 (0.92 - 2.43) | 1.46 (1 - 2.12) | 1.53 (1.22 - 1.91) | 1.26 (1.1 - 1.43) |  |
|  | 4.7% | 4.5% | 5.1% | 7.8% | 9.3% | 13.1% | 16.8% |
| **Illicit Substance** | 0.24 (0.11 - 0.5) | 0.23 (0.13 - 0.41) | 0.26 (0.15 - 0.44) | 0.4 (0.26 - 0.61) | 0.48 (0.37 - 0.61) | 0.7 (0.62 - 0.79) |  |
|  | 13.9% | 13.6% | 16.2% | 13.9% | 13.5% | 13.6% | 14.9% |
| **Pharmaceutical medications** | 0.92 (0.47 - 1.82) | 0.89 (0.51 - 1.56) | 1.11 (0.72 - 1.71) | 0.92 (0.64 - 1.32) | 0.88 (0.71 - 1.09) | 0.88 (0.78 - 0.99) |  |
|  | 3.4% | 3.1% | 3.1% | 3.4% | 3.7% | 4.5% | 4.5% |
| **Unknown substance** | 0.73 (0.42 - 1.29) | 0.67 (0.42 - 1.06) | 0.67 (0.45 - 0.99) | 0.73 (0.53 - 1.01) | 0.79 (0.64 - 0.98) | 1 (0.88 - 1.13) |  |
|  | 59.3% | 56.3% | 58.6% | 55.4% | 51.5% | 47.5% | 37.5% |
| **Self Harm^’^** | 2.48 (1.34 - 4.56) | 2.2 (1.35 - 3.57) | 2.45 (1.62 - 3.7) | 2.16 (1.58 - 2.96) | 1.89 (1.57 - 2.28) | 1.69 (1.52 - 1.87) |  |
|  | 89.4% | 88.3% | 89.2% | 88.0% | 86.2% | 84.4% | 78.5% |
| **Transport to Hospital** | 2.35 (1.61 - 3.41) | 2.11 (1.57 - 2.84) | 2.32 (1.76 - 3.04) | 2.08 (1.68 - 2.56) | 1.81 (1.58 - 2.06) | 1.62 (1.5 - 1.76) |  |
|  | 48.6% | 46.1% | 47.0% | 44.7% | 42.9% | 41.9% | 37.3% |
| **Police attended** | 1.61 (1.12 - 2.31) | 1.45 (1.08 - 1.95) | 1.52 (1.19 - 1.94) | 1.38 (1.14 - 1.68) | 1.3 (1.15 - 1.47) | 1.28 (1.19 - 1.38) |  |
| Results presented as the mean proportion of attendances and range of Odds Ratios (95%) compared to non-frequent presenter groups for each calendar year. Odds ratios calculated to account for within person clustering.  $ Area of Residence SEIFA rating - Quintile rating ( 1 to 5) of relative social disadvantage according to the patient’s postcode of residence, based on the SocioEconomic Indexes For Areas Index of Relative Socio-economic Disadvantage. A lower quintile number indicates an area of greater socioeconomic disadvantage compared to an area with a higher score  ^%^ Psychiatric symptoms - includes symptoms of anxiety, depression, psychosis, social and emotional distress or other unspecified mental health symptoms  § AOD Issues – cases where recent (in the past 24 hours) over or inappropriate use of alcohol, illicit drugs or pharmaceutical medications was a contributing reason for attendance  ^‘^ Self Harm – includes cases involving self-injury, suicidal ideation, suicide attempt or suicide  **Supplementary Table 2 - The proportion and odds ratio (95% CI) of demographic and presentation characteristics of frequent presenter groups in 2018** | | | | | | | |
| **2018** | **Frequent Mental Health Presenter Cohorts** | | | | | | **All individuals** |
| **Characteristics** | **Top 2%** | **Truncated Poisson** | **> 2 Std Dev** | **Top 5%** | **Top 10%** | **Top 20%** |  |
| **Regional location** | 29.2% | 23.3% | 25.0% | 21.6% | 22.5% | 22.4% | 24.2% |
| **Regional location** | 1.3 (0.56 - 3) | 0.95 (0.43 - 2.09) | 1.05 (0.55 - 2) | 0.86 (0.53 - 1.4) | 0.9 (0.69 - 1.18) | 0.88 (0.77 - 1.02) |  |
|  | 68.4% | 62.5% | 57.2% | 53.1% | 54.5% | 50.5% | 47.0% |
| **Female gender** | 2.48 (0.91 - 6.75) | 1.91 (0.87 - 4.18) | 1.53 (0.82 - 2.87) | 1.29 (0.84 - 1.99) | 1.4 (1.09 - 1.79) | 1.2 (1.06 - 1.36) |  |
|  |  |  |  |  |  |  |  |
| **Age group** | 17.2% | 17.7% | 18.0% | 17.4% | 18.4% | 19.4% | 25.1% |
| **15 - 24** | 0.61 (0.19 - 1.93) | 0.63 (0.24 - 1.65) | 0.64 (0.29 - 1.43) | 0.61 (0.35 - 1.08) | 0.65 (0.47 - 0.88) | 0.66 (0.57 - 0.77) |  |
|  | 48.1% | 48.4% | 45.0% | 39.8% | 41.7% | 45.6% | 45.1% |
| **25 - 44** | 1.13 (0.48 - 2.65) | 1.14 (0.55 - 2.36) | 0.99 (0.54 - 1.83) | 0.79 (0.51 - 1.23) | 0.86 (0.67 - 1.09) | 1.03 (0.91 - 1.16) |  |
|  | 34.8% | 34.0% | 37.0% | 42.8% | 39.9% | 34.9% | 29.7% |
| **45 - 65** | 1.26 (0.52 - 3.08) | 1.22 (0.57 - 2.62) | 1.4 (0.76 - 2.61) | 1.83 (1.2 - 2.79) | 1.66 (1.3 - 2.12) | 1.36 (1.19 - 1.55) |  |
|  |  |  |  |  |  |  |  |
| **Area of residence ^$^** | 42.2% | 35.9% | 31.9% | 28.1% | 25.4% | 25.0% | 24.6% |
| **SEIFA 1 ( most disadvantage)** | 2.28 (1.03 - 5.05) | 1.74 (0.86 - 3.51) | 1.46 (0.79 - 2.67) | 1.21 (0.77 - 1.89) | 1.05 (0.8 - 1.37) | 1.03 (0.9 - 1.18) |  |
|  | 17.4% | 14.5% | 14.7% | 15.5% | 15.4% | 14.6% | 15.6% |
| **SEIFA 2** | 1.15 (0.45 - 2.9) | 0.92 (0.38 - 2.21) | 0.93 (0.45 - 1.94) | 1 (0.61 - 1.64) | 0.99 (0.74 - 1.31) | 0.91 (0.78 - 1.06) |  |
|  | 13.7% | 16.9% | 18.1% | 16.8% | 15.7% | 15.7% | 16.8% |
| **SEIFA 3** | 0.78 (0.27 - 2.25) | 1.01 (0.45 - 2.27) | 1.1 (0.57 - 2.11) | 1 (0.63 - 1.61) | 0.92 (0.69 - 1.22) | 0.9 (0.78 - 1.05) |  |
|  | 11.7% | 13.8% | 14.0% | 16.5% | 17.9% | 17.2% | 17.2% |
| **SEIFA 4** | 0.63 (0.23 - 1.74) | 0.77 (0.35 - 1.7) | 0.78 (0.4 - 1.52) | 0.95 (0.61 - 1.47) | 1.05 (0.82 - 1.35) | 1 (0.88 - 1.15) |  |
|  | 3.2% | 3.6% | 6.3% | 9.3% | 12.5% | 14.5% | 16.3% |
| **SEIFA 5 (least disadvantage)** | 0.16 (0.04 - 0.7) | 0.19 (0.06 - 0.55) | 0.34 (0.15 - 0.75) | 0.51 (0.31 - 0.83) | 0.72 (0.55 - 0.93) | 0.84 (0.74 - 0.96) |  |
|  | 11.8% | 15.3% | 14.9% | 13.8% | 12.9% | 12.9% | 8.5% |
| **Residence not recorded** | 1.47 | 2.00 | 1.94 | 1.79 | 1.72 | 1.90 |  |
|  | 5.0% | 6.2% | 5.8% | 6.0% | 5.5% | 5.6% | 3.1% |
| **Housing Problems** | 1.68 | 2.14 | 2.02 | 2.13 | 2.04 | 2.46 |  |
|  |  |  |  |  |  |  |  |
| **Time of the day** | 27.0% | 28.3% | 27.7% | 28.4% | 30.5% | 31.1% | 29.4% |
| **Day (08:00 - 16:59)** | 0.89 (0.6 - 1.31) | 0.95 (0.69 - 1.29) | 0.92 (0.71 - 1.19) | 0.95 (0.79 - 1.15) | 1.06 (0.95 - 1.18) | 1.11 (1.05 - 1.18) |  |
|  | 53.4% | 53.2% | 53.5% | 53.6% | 52.2% | 50.8% | 47.0% |
| **Evening (17:00 - 23:59)** | 1.3 (1.01 - 1.68) | 1.29 (1.05 - 1.6) | 1.31 (1.1 - 1.56) | 1.32 (1.16 - 1.51) | 1.26 (1.16 - 1.37) | 1.22 (1.16 - 1.28) |  |
|  | 19.6% | 18.4% | 18.7% | 17.8% | 17.3% | 18.0% | 23.6% |
| **Night (00:00 - 07:59)** | 0.78 (0.48 - 1.28) | 0.73 (0.48 - 1.11) | 0.74 (0.52 - 1.04) | 0.69 (0.54 - 0.88) | 0.65 (0.56 - 0.75) | 0.65 (0.6 - 0.71) |  |
|  | 28.9% | 28.2% | 27.3% | 27.3% | 27.9% | 27.7% | 32.7% |
| **Weekend attendance** | 0.83 (0.72 - 0.97) | 0.8 (0.7 - 0.92) | 0.77 (0.68 - 0.86) | 0.76 (0.69 - 0.84) | 0.78 (0.73 - 0.83) | 0.74 (0.71 - 0.77) |  |
|  | 21.2% | 21.5% | 21.9% | 19.4% | 26.4% | 31.6% | 34.2% |
| **Psychiatric symptoms ^%^** | 0.51 (0.3 - 0.87) | 0.52 (0.34 - 0.8) | 0.53 (0.37 - 0.77) | 0.45 (0.34 - 0.59) | 0.67 (0.58 - 0.77) | 0.86 (0.8 - 0.93) |  |
|  | 0.8% | 0.8% | 0.9% | 1.3% | 3.7% | 5.7% | 10.3% |
| **Anxiety** | 0.07 (0.04 - 0.14) | 0.07 (0.04 - 0.13) | 0.08 (0.05 - 0.13) | 0.11 (0.08 - 0.15) | 0.31 (0.24 - 0.39) | 0.46 (0.41 - 0.52) |  |
|  | 4.1% | 4.3% | 5.1% | 4.5% | 4.7% | 5.1% | 5.5% |
| **Depression** | 0.74 (0.43 - 1.26) | 0.77 (0.48 - 1.23) | 0.92 (0.63 - 1.35) | 0.81 (0.6 - 1.08) | 0.83 (0.7 - 0.99) | 0.89 (0.8 - 0.99) |  |
|  | 11.6% | 10.4% | 9.7% | 7.6% | 8.9% | 9.3% | 7.1% |
| **Psychosis Symptoms** | 1.73 (0.75 - 3.96) | 1.54 (0.74 - 3.19) | 1.41 (0.75 - 2.66) | 1.08 (0.65 - 1.81) | 1.31 (1.01 - 1.7) | 1.48 (1.3 - 1.69) |  |
|  | 5.1% | 6.4% | 6.8% | 6.4% | 9.7% | 12.4% | 12.0% |
| **Other MH Sx** | 0.39 (0.19 - 0.78) | 0.49 (0.28 - 0.86) | 0.52 (0.32 - 0.86) | 0.49 (0.34 - 0.7) | 0.77 (0.64 - 0.92) | 1.04 (0.95 - 1.14) |  |
|  | 56.8% | 57.5% | 57.2% | 62.4% | 60.4% | 60.2% | 61.1% |
| **AOD issues** ^§^ | 0.83 (0.46 - 1.53) | 0.86 (0.52 - 1.42) | 0.85 (0.55 - 1.31) | 1.06 (0.77 - 1.46) | 0.97 (0.81 - 1.16) | 0.95 (0.87 - 1.05) |  |
|  | 35.0% | 34.7% | 38.2% | 44.3% | 40.1% | 34.6% | 33.6% |
| **Alcohol** | 1.07 (0.51 - 2.24) | 1.05 (0.57 - 1.96) | 1.23 (0.73 - 2.06) | 1.62 (1.13 - 2.31) | 1.37 (1.11 - 1.68) | 1.06 (0.95 - 1.18) |  |
|  | 10.1% | 9.6% | 7.8% | 7.6% | 10.0% | 14.0% | 16.9% |
| **Illicit Substance** | 0.55 (0.33 - 0.92) | 0.51 (0.32 - 0.81) | 0.41 (0.26 - 0.65) | 0.39 (0.26 - 0.59) | 0.52 (0.42 - 0.64) | 0.75 (0.68 - 0.83) |  |
|  | 20.6% | 20.0% | 17.1% | 15.8% | 14.7% | 15.4% | 14.8% |
| **Pharmaceutical medications** | 1.51 (0.77 - 2.95) | 1.45 (0.82 - 2.59) | 1.19 (0.7 - 2.01) | 1.09 (0.74 - 1.59) | 0.99 (0.79 - 1.24) | 1.06 (0.95 - 1.19) |  |
|  | 3.5% | 3.6% | 2.9% | 2.7% | 3.1% | 4.3% | 4.5% |
| **Unknown substance** | 0.77 (0.39 - 1.55) | 0.78 (0.44 - 1.4) | 0.64 (0.36 - 1.13) | 0.58 (0.38 - 0.89) | 0.65 (0.51 - 0.84) | 0.94 (0.84 - 1.06) |  |
|  | 71.2% | 68.5% | 64.5% | 57.6% | 51.5% | 46.3% | 37.1% |
| **Self Harm^’^** | 4.31 (2.31 - 8.04) | 3.8 (2.29 - 6.31) | 3.19 (2.09 - 4.86) | 2.4 (1.78 - 3.24) | 1.93 (1.62 - 2.3) | 1.63 (1.48 - 1.79) |  |
|  | 92.3% | 92.6% | 91.8% | 89.7% | 89.0% | 87.1% | 81.4% |
| **Transport to Hospital** | 2.78 (1.88 - 4.11) | 2.92 (2.1 - 4.07) | 2.6 (1.94 - 3.49) | 2.05 (1.63 - 2.58) | 1.95 (1.69 - 2.25) | 1.72 (1.59 - 1.86) |  |
|  | 51.2% | 52.0% | 50.1% | 46.6% | 43.4% | 43.0% | 39.0% |
| **Police attended** | 1.65 (1.17 - 2.34) | 1.71 (1.28 - 2.3) | 1.59 (1.23 - 2.06) | 1.39 (1.15 - 1.67) | 1.22 (1.09 - 1.37) | 1.24 (1.16 - 1.32) |  |
| Results presented as the mean proportion of attendances and range of Odds Ratios (95%) compared to non-frequent presenter groups for each calendar year. Odds ratios calculated to account for within person clustering.  $ Area of Residence SEIFA rating - Quintile rating ( 1 to 5) of relative social disadvantage according to the patient’s postcode of residence, based on the SocioEconomic Indexes For Areas Index of Relative Socio-economic Disadvantage. A lower quintile number indicates an area of greater socioeconomic disadvantage compared to an area with a higher score  ^%^ Psychiatric symptoms - includes symptoms of anxiety, depression, psychosis, social and emotional distress or other unspecified mental health symptoms  § AOD Issues – cases where recent (in the past 24 hours) over or inappropriate use of alcohol, illicit drugs or pharmaceutical medications was a contributing reason for attendance  ^‘^ Self Harm – includes cases involving self-injury, suicidal ideation, suicide attempt or suicide  **Supplementary Table 3 - The proportion and odds ratio (95% CI) of demographic and presentation characteristics of frequent presenter groups in 2019** | | | | | | | |
| **2019** | **Frequent Mental Health Presenter Cohorts** | | | | | | **All individuals** |
| **Characteristics** | **Top 2%** | **Truncated Poisson** | **> 2 Std Dev** | **Top 5%** | **Top 10%** | **Top 20%** |  |
| **Regional location** | 23.2% | 20.9% | 21.1% | 19.3% | 20.5% | 21.5% | 24.5% |
| **Regional location** | 0.93 (0.41 - 2.1) | 0.81 (0.4 - 1.63) | 0.82 (0.46 - 1.46) | 0.73 (0.46 - 1.13) | 0.77 (0.6 - 0.99) | 0.81 (0.71 - 0.91) |  |
|  | 65.7% | 66.0% | 62.5% | 58.5% | 52.6% | 48.5% | 46.3% |
| **Female gender** | 2.26 (1.02 - 5.02) | 2.3 (1.21 - 4.4) | 1.98 (1.18 - 3.33) | 1.68 (1.16 - 2.45) | 1.33 (1.07 - 1.64) | 1.13 (1.01 - 1.26) |  |
|  |  |  |  |  |  |  |  |
| **Age group** | 24.2% | 26.4% | 24.0% | 23.6% | 20.9% | 20.5% | 25.1% |
| **15 - 24** | 0.95 (0.41 - 2.22) | 1.07 (0.55 - 2.09) | 0.94 (0.53 - 1.68) | 0.92 (0.6 - 1.41) | 0.77 (0.59 - 1) | 0.72 (0.62 - 0.82) |  |
|  | 40.6% | 40.4% | 37.5% | 38.7% | 42.0% | 45.9% | 45.4% |
| **25 - 44** | 0.82 (0.38 - 1.77) | 0.81 (0.43 - 1.52) | 0.72 (0.42 - 1.22) | 0.75 (0.51 - 1.1) | 0.86 (0.69 - 1.06) | 1.03 (0.92 - 1.15) |  |
|  | 35.2% | 33.2% | 38.4% | 37.7% | 37.1% | 33.6% | 29.5% |
| **45 - 65** | 1.3 (0.6 - 2.84) | 1.19 (0.62 - 2.27) | 1.51 (0.91 - 2.53) | 1.48 (1.01 - 2.16) | 1.47 (1.18 - 1.83) | 1.28 (1.14 - 1.44) |  |
|  |  |  |  |  |  |  |  |
| **Area of residence ^$^** | 36.7% | 37.1% | 33.2% | 27.3% | 25.6% | 24.8% | 24.4% |
| **SEIFA 1 ( most disadvantage)** | 1.82 (0.85 - 3.88) | 1.86 (1.03 - 3.38) | 1.57 (0.93 - 2.63) | 1.17 (0.77 - 1.77) | 1.07 (0.84 - 1.37) | 1.03 (0.91 - 1.17) |  |
|  | 14.8% | 15.9% | 17.3% | 16.5% | 15.4% | 15.6% | 15.7% |
| **SEIFA 2** | 0.93 (0.39 - 2.25) | 1.02 (0.51 - 2.04) | 1.13 (0.65 - 1.96) | 1.06 (0.7 - 1.62) | 0.98 (0.76 - 1.26) | 0.99 (0.87 - 1.13) |  |
|  | 10.0% | 11.8% | 12.6% | 13.0% | 13.3% | 14.4% | 16.4% |
| **SEIFA 3** | 0.56 (0.2 - 1.55) | 0.68 (0.32 - 1.45) | 0.73 (0.39 - 1.35) | 0.75 (0.48 - 1.18) | 0.76 (0.59 - 0.99) | 0.83 (0.73 - 0.94) |  |
|  | 10.5% | 8.5% | 10.4% | 13.3% | 15.2% | 15.8% | 17.0% |
| **SEIFA 4** | 0.57 (0.22 - 1.48) | 0.44 (0.18 - 1.07) | 0.56 (0.29 - 1.07) | 0.74 (0.48 - 1.12) | 0.86 (0.68 - 1.09) | 0.89 (0.79 - 1.01) |  |
|  | 8.4% | 8.2% | 9.0% | 13.5% | 14.7% | 15.0% | 16.1% |
| **SEIFA 5 (least disadvantage)** | 0.47 (0.19 - 1.17) | 0.46 (0.21 - 0.99) | 0.5 (0.27 - 0.94) | 0.8 (0.55 - 1.18) | 0.89 (0.71 - 1.11) | 0.9 (0.8 - 1.01) |  |
|  | 18.7% | 17.7% | 17.0% | 16.1% | 15.3% | 14.0% | 9.2% |
| **Residence not recorded** | 2.33 (1.16 - 4.68) | 2.2 (1.25 - 3.86) | 2.1 (1.31 - 3.37) | 1.99 (1.39 - 2.84) | 1.96 (1.59 - 2.41) | 1.95 (1.73 - 2.19) |  |
|  | 8.3% | 7.8% | 7.2% | 7.0% | 7.2% | 6.4% | 3.6% |
| **Housing Problems** | 2.49 (1.35 - 4.6) | 2.35 (1.41 - 3.92) | 2.17 (1.37 - 3.43) | 2.13 (1.5 - 3.02) | 2.36 (1.92 - 2.9) | 2.42 (2.13 - 2.74) |  |
|  |  |  |  |  |  |  |  |
| **Time of the day** | 23.9% | 25.4% | 27.8% | 27.2% | 29.0% | 31.3% | 29.3% |
| **Day (08:00 - 16:59)** | 0.75 (0.54 - 1.04) | 0.82 (0.63 - 1.06) | 0.93 (0.74 - 1.16) | 0.9 (0.76 - 1.07) | 0.98 (0.89 - 1.08) | 1.13 (1.07 - 1.19) |  |
|  | 54.9% | 54.0% | 51.9% | 52.9% | 51.5% | 49.3% | 46.5% |
| **Evening (17:00 - 23:59)** | 1.41 (1.11 - 1.78) | 1.36 (1.12 - 1.65) | 1.25 (1.05 - 1.48) | 1.31 (1.15 - 1.49) | 1.25 (1.16 - 1.35) | 1.15 (1.1 - 1.21) |  |
|  | 21.3% | 20.6% | 20.2% | 19.8% | 19.4% | 19.3% | 24.1% |
| **Night (00:00 - 07:59)** | 0.85 (0.58 - 1.25) | 0.81 (0.59 - 1.11) | 0.79 (0.6 - 1.04) | 0.77 (0.63 - 0.94) | 0.74 (0.65 - 0.83) | 0.7 (0.66 - 0.75) |  |
|  | 27.7% | 27.7% | 27.4% | 26.8% | 26.3% | 26.8% | 32.3% |
| **Weekend attendance** | 0.8 (0.72 - 0.89) | 0.8 (0.73 - 0.88) | 0.79 (0.72 - 0.86) | 0.76 (0.7 - 0.81) | 0.72 (0.69 - 0.76) | 0.71 (0.69 - 0.74) |  |
|  | 22.8% | 25.2% | 23.3% | 22.1% | 26.2% | 33.3% | 34.8% |
| **Psychiatric symptoms ^%^** | 0.55 (0.36 - 0.83) | 0.63 (0.44 - 0.88) | 0.56 (0.42 - 0.75) | 0.52 (0.41 - 0.64) | 0.64 (0.56 - 0.72) | 0.92 (0.86 - 0.98) |  |
|  | 1.9% | 2.1% | 2.4% | 2.9% | 3.6% | 5.0% | 9.3% |
| **Anxiety** | 0.19 (0.11 - 0.31) | 0.2 (0.13 - 0.31) | 0.23 (0.15 - 0.36) | 0.28 (0.19 - 0.42) | 0.33 (0.26 - 0.42) | 0.45 (0.4 - 0.51) |  |
|  | 3.6% | 3.4% | 3.4% | 4.4% | 4.5% | 5.0% | 5.6% |
| **Depression** | 0.61 (0.39 - 0.95) | 0.59 (0.41 - 0.85) | 0.57 (0.42 - 0.78) | 0.77 (0.61 - 0.96) | 0.78 (0.67 - 0.91) | 0.85 (0.77 - 0.93) |  |
|  | 11.0% | 10.9% | 9.4% | 7.8% | 8.2% | 9.9% | 7.8% |
| **Psychosis Symptoms** | 1.49 (0.79 - 2.8) | 1.47 (0.88 - 2.45) | 1.24 (0.77 - 1.98) | 1 (0.68 - 1.48) | 1.07 (0.86 - 1.33) | 1.43 (1.29 - 1.59) |  |
|  | 6.6% | 9.3% | 8.6% | 7.3% | 10.4% | 14.2% | 12.9% |
| **Other MH Sx** | 0.47 (0.32 - 0.71) | 0.68 (0.46 - 1.02) | 0.62 (0.44 - 0.89) | 0.52 (0.39 - 0.69) | 0.77 (0.66 - 0.89) | 1.15 (1.06 - 1.25) |  |
|  | 54.5% | 55.2% | 57.9% | 61.0% | 62.3% | 60.4% | 61.2% |
| **AOD issues** ^§^ | 0.75 (0.46 - 1.25) | 0.78 (0.52 - 1.16) | 0.87 (0.62 - 1.22) | 0.99 (0.77 - 1.28) | 1.06 (0.9 - 1.23) | 0.96 (0.89 - 1.04) |  |
|  | 30.8% | 29.3% | 34.4% | 39.6% | 40.4% | 34.9% | 33.9% |
| **Alcohol** | 0.86 (0.43 - 1.74) | 0.8 (0.45 - 1.42) | 1.02 (0.65 - 1.59) | 1.29 (0.94 - 1.77) | 1.36 (1.13 - 1.64) | 1.05 (0.95 - 1.16) |  |
|  | 6.8% | 6.8% | 6.9% | 7.3% | 10.1% | 14.3% | 17.5% |
| **Illicit Substance** | 0.34 (0.17 - 0.66) | 0.34 (0.2 - 0.58) | 0.34 (0.22 - 0.54) | 0.36 (0.26 - 0.5) | 0.5 (0.42 - 0.6) | 0.74 (0.68 - 0.81) |  |
|  | 21.1% | 22.7% | 19.5% | 17.4% | 14.8% | 14.0% | 14.0% |
| **Pharmaceutical medications** | 1.65 (0.93 - 2.94) | 1.84 (1.18 - 2.87) | 1.51 (1.01 - 2.26) | 1.31 (0.96 - 1.78) | 1.07 (0.88 - 1.31) | 1 (0.9 - 1.11) |  |
|  | 2.9% | 3.4% | 3.7% | 3.1% | 3.6% | 4.3% | 4.3% |
| **Unknown substance** | 0.65 (0.4 - 1.05) | 0.77 (0.52 - 1.14) | 0.85 (0.61 - 1.19) | 0.7 (0.53 - 0.94) | 0.81 (0.67 - 0.97) | 1 (0.9 - 1.11) |  |
|  | 67.9% | 65.4% | 62.0% | 59.0% | 51.1% | 45.2% | 37.0% |
| **Self Harm^’^** | 3.7 (2.22 - 6.15) | 3.32 (2.19 - 5.05) | 2.89 (2.04 - 4.09) | 2.58 (1.99 - 3.34) | 1.91 (1.63 - 2.23) | 1.56 (1.43 - 1.7) |  |
|  | 91.0% | 91.1% | 90.9% | 89.9% | 89.2% | 88.4% | 83.5% |
| **Transport to Hospital** | 2.02 (1.52 - 2.68) | 2.04 (1.61 - 2.58) | 2.01 (1.62 - 2.49) | 1.8 (1.49 - 2.16) | 1.71 (1.52 - 1.93) | 1.67 (1.55 - 1.79) |  |
|  | 51.4% | 51.1% | 49.7% | 47.2% | 45.6% | 45.8% | 41.1% |
| **Police attended** | 1.53 (1.1 - 2.13) | 1.52 (1.15 - 2) | 1.44 (1.13 - 1.83) | 1.3 (1.08 - 1.57) | 1.23 (1.1 - 1.37) | 1.28 (1.2 - 1.36) |  |
| Results presented as the mean proportion of attendances and range of Odds Ratios (95%) compared to non-frequent presenter groups for each calendar year. Odds ratios calculated to account for within person clustering.  $ Area of Residence SEIFA rating - Quintile rating ( 1 to 5) of relative social disadvantage according to the patient’s postcode of residence, based on the SocioEconomic Indexes For Areas Index of Relative Socio-economic Disadvantage. A lower quintile number indicates an area of greater socioeconomic disadvantage compared to an area with a higher score  ^%^ Psychiatric symptoms - includes symptoms of anxiety, depression, psychosis, social and emotional distress or other unspecified mental health symptoms  § AOD Issues – cases where recent (in the past 24 hours) over or inappropriate use of alcohol, illicit drugs or pharmaceutical medications was a contributing reason for attendance  ^‘^ Self Harm – includes cases involving self-injury, suicidal ideation, suicide attempt or suicide    **Supplementary Table 4 - The proportion and odds ratio (95% CI) of demographic and presentation characteristics of frequent presenter groups in 2020** | | | | | | | |
| **2020** | **Frequent Mental Health Presenter Cohorts** | | | | | | **All individuals** |
| **Characteristics** | **Top 2%** | **Truncated Poisson** | **> 2 Std Dev** | **Top 5%** | **Top 10%** | **Top 20%** |  |
| **Regional location** | 8.2% | 13.6% | 13.6% | 13.4% | 17.4% | 19.7% | 25.1% |
| **Regional location** | 0.26 (0.08 - 0.84) | 0.46 (0.24 - 0.89) | 0.46 (0.25 - 0.86) | 0.45 (0.27 - 0.74) | 0.6 (0.46 - 0.77) | 0.68 (0.6 - 0.78) |  |
|  | 69.3% | 71.4% | 69.7% | 65.5% | 56.5% | 51.9% | 47.1% |
| **Female gender** | 2.59 (1.19 - 5.64) | 2.9 (1.61 - 5.22) | 2.67 (1.55 - 4.6) | 2.22 (1.47 - 3.35) | 1.53 (1.22 - 1.91) | 1.28 (1.13 - 1.45) |  |
|  |  |  |  |  |  |  |  |
| **Age group** | 27.6% | 32.3% | 35.9% | 31.0% | 28.1% | 24.4% | 25.2% |
| **15 - 24** | 1.14 (0.48 - 2.69) | 1.43 (0.8 - 2.57) | 1.7 (1 - 2.89) | 1.36 (0.87 - 2.1) | 1.18 (0.91 - 1.53) | 0.95 (0.81 - 1.11) |  |
|  | 37.7% | 39.8% | 37.4% | 40.6% | 42.8% | 46.0% | 46.1% |
| **25 - 44** | 0.7 (0.34 - 1.44) | 0.76 (0.45 - 1.3) | 0.69 (0.41 - 1.14) | 0.79 (0.53 - 1.17) | 0.86 (0.69 - 1.07) | 0.99 (0.88 - 1.12) |  |
|  | 34.6% | 27.9% | 26.7% | 28.3% | 29.1% | 29.6% | 28.6% |
| **45 - 65** | 1.33 (0.6 - 2.92) | 0.96 (0.51 - 1.81) | 0.91 (0.49 - 1.66) | 0.99 (0.62 - 1.55) | 1.03 (0.8 - 1.33) | 1.06 (0.93 - 1.22) |  |
|  |  |  |  |  |  |  |  |
| **Area of residence ^$^** | 37.1% | 34.4% | 34.8% | 33.4% | 27.5% | 25.2% | 25.0% |
| **SEIFA 1 ( most disadvantage)** | 1.8 (0.89 - 3.64) | 1.6 (0.95 - 2.71) | 1.64 (1 - 2.67) | 1.54 (1.05 - 2.27) | 1.16 (0.91 - 1.48) | 1.02 (0.88 - 1.17) |  |
|  | 11.8% | 17.2% | 17.4% | 16.2% | 17.2% | 16.6% | 16.4% |
| **SEIFA 2** | 0.68 (0.32 - 1.43) | 1.06 (0.62 - 1.79) | 1.07 (0.66 - 1.75) | 0.98 (0.65 - 1.47) | 1.06 (0.84 - 1.34) | 1.02 (0.89 - 1.16) |  |
|  | 12.7% | 10.5% | 10.3% | 11.2% | 12.8% | 13.6% | 16.3% |
| **SEIFA 3** | 0.74 (0.29 - 1.88) | 0.59 (0.29 - 1.24) | 0.58 (0.29 - 1.17) | 0.63 (0.37 - 1.07) | 0.73 (0.55 - 0.96) | 0.77 (0.66 - 0.89) |  |
|  | 16.1% | 16.1% | 15.2% | 13.5% | 15.6% | 16.9% | 17.0% |
| **SEIFA 4** | 0.93 (0.37 - 2.36) | 0.93 (0.48 - 1.81) | 0.87 (0.46 - 1.65) | 0.75 (0.44 - 1.29) | 0.89 (0.67 - 1.18) | 0.98 (0.85 - 1.14) |  |
|  | 14.0% | 13.8% | 13.8% | 17.0% | 16.2% | 15.9% | 16.7% |
| **SEIFA 5 (least disadvantage)** | 0.81 (0.33 - 2.01) | 0.79 (0.41 - 1.55) | 0.79 (0.42 - 1.47) | 1.02 (0.65 - 1.59) | 0.96 (0.74 - 1.25) | 0.93 (0.8 - 1.08) |  |
|  | 8.2% | 8.0% | 8.4% | 8.7% | 10.5% | 11.6% | 7.8% |
| **Residence not recorded** | 1.05 | 1.02 | 1.09 | 1.13 | 1.45 | 1.79 |  |
|  | 4.6% | 4.6% | 4.8% | 5.0% | 6.0% | 6.6% | 4.0% |
| **Housing Problems** | 1.17 | 1.18 | 1.22 | 1.31 | 1.66 | 2.11 |  |
|  |  |  |  |  |  |  |  |
| **Time of the day** | 25.7% | 26.9% | 26.4% | 26.1% | 28.1% | 30.0% | 29.9% |
| **Day (08:00 - 16:59)** | 0.81 (0.61 - 1.07) | 0.86 (0.71 - 1.04) | 0.84 (0.69 - 1.01) | 0.82 (0.71 - 0.95) | 0.91 (0.83 - 0.99) | 1.01 (0.96 - 1.07) |  |
|  | 55.8% | 57.4% | 57.3% | 57.2% | 54.0% | 50.9% | 47.2% |
| **Evening (17:00 - 23:59)** | 1.42 (1.17 - 1.72) | 1.53 (1.32 - 1.76) | 1.52 (1.33 - 1.74) | 1.53 (1.36 - 1.71) | 1.36 (1.26 - 1.46) | 1.21 (1.15 - 1.26) |  |
|  | 18.5% | 15.7% | 16.3% | 16.6% | 17.8% | 18.9% | 22.8% |
| **Night (00:00 - 07:59)** | 0.76 (0.58 - 1) | 0.62 (0.49 - 0.78) | 0.65 (0.52 - 0.81) | 0.66 (0.55 - 0.79) | 0.71 (0.63 - 0.79) | 0.75 (0.7 - 0.8) |  |
|  | 30.5% | 29.6% | 29.4% | 28.4% | 27.9% | 27.6% | 30.8% |
| **Weekend attendance** | 0.99 (0.86 - 1.14) | 0.94 (0.84 - 1.06) | 0.93 (0.84 - 1.04) | 0.89 (0.82 - 0.97) | 0.86 (0.81 - 0.91) | 0.82 (0.79 - 0.86) |  |
|  | 31.2% | 26.6% | 26.9% | 28.8% | 30.8% | 35.1% | 38.3% |
| **Psychiatric symptoms ^%^** | 0.73 (0.49 - 1.09) | 0.58 (0.42 - 0.79) | 0.58 (0.43 - 0.78) | 0.64 (0.51 - 0.81) | 0.69 (0.61 - 0.79) | 0.84 (0.78 - 0.91) |  |
|  | 4.1% | 3.3% | 3.3% | 3.6% | 4.3% | 5.6% | 9.9% |
| **Anxiety** | 0.39 (0.12 - 1.21) | 0.3 (0.12 - 0.76) | 0.3 (0.13 - 0.71) | 0.33 (0.18 - 0.63) | 0.38 (0.28 - 0.53) | 0.48 (0.42 - 0.56) |  |
|  | 4.6% | 4.0% | 3.9% | 4.1% | 4.5% | 4.8% | 5.5% |
| **Depression** | 0.83 (0.54 - 1.27) | 0.71 (0.49 - 1.02) | 0.69 (0.49 - 0.98) | 0.73 (0.55 - 0.96) | 0.79 (0.67 - 0.92) | 0.84 (0.76 - 0.93) |  |
|  | 15.2% | 12.3% | 11.9% | 12.5% | 11.2% | 11.0% | 8.7% |
| **Psychosis Symptoms** | 1.91 (1.02 - 3.59) | 1.48 (0.88 - 2.49) | 1.43 (0.87 - 2.35) | 1.53 (1.05 - 2.22) | 1.36 (1.09 - 1.71) | 1.39 (1.22 - 1.58) |  |
|  | 8.7% | 8.4% | 9.0% | 9.7% | 12.0% | 15.2% | 15.6% |
| **Other MH Sx** | 0.51 (0.3 - 0.88) | 0.48 (0.33 - 0.7) | 0.52 (0.36 - 0.76) | 0.57 (0.42 - 0.77) | 0.72 (0.61 - 0.85) | 0.96 (0.88 - 1.05) |  |
|  | 46.2% | 50.0% | 50.5% | 52.1% | 57.3% | 58.1% | 59.1% |
| **AOD issues** ^§^ | 1.06 (0.98 - 1.15) | 0.59 (0.36 - 0.95) | 0.68 (0.48 - 0.98) | 0.69 (0.49 - 0.98) | 0.74 (0.56 - 0.98) | 0.92 (0.78 - 1.08) |  |
|  | 26.4% | 29.0% | 29.3% | 34.1% | 36.3% | 33.9% | 31.4% |
| **Alcohol** | 0.78 (0.39 - 1.57) | 0.89 (0.54 - 1.47) | 0.9 (0.56 - 1.44) | 1.14 (0.8 - 1.62) | 1.28 (1.05 - 1.56) | 1.16 (1.03 - 1.29) |  |
|  | 5.6% | 6.2% | 6.8% | 6.0% | 10.7% | 15.0% | 18.8% |
| **Illicit Substance** | 0.25 (0.13 - 0.49) | 0.28 (0.16 - 0.47) | 0.31 (0.19 - 0.5) | 0.27 (0.18 - 0.4) | 0.49 (0.4 - 0.6) | 0.71 (0.64 - 0.79) |  |
|  | 13.7% | 14.7% | 14.6% | 12.7% | 12.7% | 12.6% | 13.6% |
| **Pharmaceutical medications** | 1 (0.59 - 1.71) | 1.09 (0.75 - 1.58) | 1.09 (0.76 - 1.55) | 0.92 (0.68 - 1.24) | 0.91 (0.76 - 1.08) | 0.89 (0.8 - 0.98) |  |
|  | 2.8% | 2.5% | 2.6% | 2.0% | 3.0% | 3.8% | 4.1% |
| **Unknown substance** | 0.66 (0.36 - 1.21) | 0.59 (0.37 - 0.94) | 0.61 (0.4 - 0.93) | 0.47 (0.32 - 0.69) | 0.69 (0.56 - 0.85) | 0.89 (0.79 - 1) |  |
|  | 67.5% | 66.9% | 66.2% | 61.4% | 53.0% | 46.5% | 37.4% |
| **Self Harm^’^** | 3.58 (2 - 6.41) | 3.53 (2.29 - 5.47) | 3.44 (2.29 - 5.17) | 2.82 (2.07 - 3.83) | 2.04 (1.71 - 2.42) | 1.62 (1.46 - 1.78) |  |
|  | 90.1% | 91.4% | 91.4% | 90.7% | 90.5% | 89.4% | 84.9% |
| **Transport to Hospital** | 1.63 (1.05 - 2.53) | 1.92 (1.35 - 2.72) | 1.93 (1.39 - 2.69) | 1.78 (1.37 - 2.3) | 1.78 (1.53 - 2.07) | 1.65 (1.52 - 1.79) |  |
|  | 62.7% | 59.8% | 59.5% | 56.4% | 52.6% | 51.6% | 45.9% |
| **Police attended** | 2.01 (1.33 - 3.03) | 1.79 (1.32 - 2.43) | 1.77 (1.33 - 2.35) | 1.56 (1.24 - 1.96) | 1.35 (1.19 - 1.54) | 1.34 (1.24 - 1.44) |  |

Results presented as the mean proportion of attendances and range of Odds Ratios (95%) compared to non-frequent presenter groups for each calendar year. Odds ratios calculated to account for within person clustering.

$ Area of Residence SEIFA rating - Quintile rating ( 1 to 5) of relative social disadvantage according to the patient’s postcode of residence, based on the SocioEconomic Indexes For Areas Index of Relative Socio-economic Disadvantage. A lower quintile number indicates an area of greater socioeconomic disadvantage compared to an area with a higher score

^%^ Psychiatric symptoms - includes symptoms of anxiety, depression, psychosis, social and emotional distress or other unspecified mental health symptoms

§ AOD Issues – cases where recent (in the past 24 hours) over or inappropriate use of alcohol, illicit drugs or pharmaceutical medications was a contributing reason for attendance

^‘^ Self Harm – includes cases involving self-injury, suicidal ideation, suicide attempt or suicide
